# Comparative Outcomes of Endoscopic Treatment for Symptomatic and Asymptomatic Common Bile Duct Stones in the Elderly: A Propensity Score‐Based Cohort Analysis

**DOI:** 10.1002/jhbp.70059

**Published:** 2025-12-30

**Authors:** Kota Shimojo, Akinori Maruta, Keisuke Iwata, Yuhei Iwasa, Mitsuru Okuno, Kensaku Yoshida, Shinya Uemura, Tsuyoshi Mukai, Takuji Iwashita, Masahito Shimizu

**Affiliations:** ^1^ Department of Gastroenterology Hashima Municipal Hospital Gifu Japan; ^2^ First Department of Internal Medicine Gifu University Hospital Gifu Gifu Japan; ^3^ Department of Gastroenterology Gifu Municipal Hospital Gifu Gifu Japan; ^4^ Department of Gastroenterology Gifu Prefectural General Medical Center Gifu Gifu Japan; ^5^ Department of Gastroenterology Shiga University of Medical Science Shiga Japan

**Keywords:** asymptomatic common bile duct stone, elderly patients, endoscopic retrograde cholangiopancreatography, post‐ERCP pancreatitis, recurrence of common bile duct stones

## Abstract

**Introduction:**

Various guidelines recommend endoscopic stone removal for asymptomatic common bile duct stones (CBDS); however, previous studies have indicated that asymptomatic CBDS is associated with a higher incidence of post‐ERCP pancreatitis (PEP). Our study aimed to compare the clinical outcomes of ERCP between elderly patients with asymptomatic and symptomatic CBDS.

**Methods:**

Elderly patients with CBDS were divided into two groups: A (asymptomatic CBDS) and S (symptomatic CBDS). Propensity score matching was performed to reduce possible bias in the baseline characteristics between the two groups, yielding 221 matched patients. Primary outcomes included early adverse events (AEs) during ERCP, while secondary outcomes included technical success rate and late AEs.

**Results:**

Regarding ERCP‐related early AEs, the incidence of PEP was 6.3% in group A and 4.5% in group S, with no significant difference between the groups. The technical success rates were 87.8% and 84.6% in groups A and S, respectively, with no significant difference. The most common late AE was the recurrence of CBDS (11.3% in group A versus 18.6% in group S [*p* = 0.04]); Kaplan–Meier analysis also revealed a tendency toward a higher cumulative incidence in group S.

**Conclusion:**

Endoscopic transpapillary stone extraction in asymptomatic elderly patients may be beneficial.

## Graphical abstract

Shimojo and colleagues retrospectively compared clinical outcomes of endoscopic treatment for asymptomatic and symptomatic common bile duct stones in patients aged ≥ 75 years using propensity score matching. Post‐ERCP pancreatitis rates did not differ significantly between groups, whereas the cumulative incidence of stone recurrence tended to be higher in symptomatic cases.

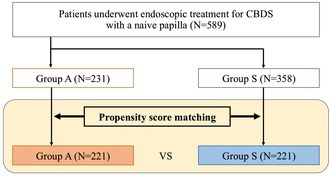

## 1 Introduction

Common bile duct stones (CBDS) are a common disease that can lead to complications including cholangitis, obstructive jaundice, and biliary pancreatitis. In addition to imaging modalities, such as transabdominal ultrasound, computed tomography, and magnetic resonance imaging, the widespread use of endoscopic ultrasound has increased the chances of detecting asymptomatic CBDS. Asymptomatic CBDS can be fatal if it becomes severe due to complications such as acute cholangitis or pancreatitis. Accordingly, the 2021 cholelithiasis guidelines suggest that stone extraction be performed for asymptomatic CBDS [1]. Similarly, various other guidelines recommend stone removal regardless of the presence or absence of symptoms [2, 3, 4]. However, several studies have reported that endoscopic retrograde cholangiopancreatography (ERCP) for asymptomatic CBDS is associated with a high incidence of post‐ERCP pancreatitis (PEP) [5, 6, 7]. Elderly patients often have poor performance status or comorbidities, such as cardiovascular disease and chronic kidney disease, which may affect the clinical course of adverse events (AEs) [8, 9]. Therefore, whether ERCP should be performed aggressively in those with asymptomatic CBDS, particularly elderly patients, remains controversial. Our study compared the clinical outcomes of ERCP for symptomatic and asymptomatic CBDS in elderly patients.

## 2 Methods

### 2.1 Study Design and Patient Selection

This retrospective multicenter cohort study was conducted at three tertiary care centers: Gifu Municipal Hospital, Gifu Prefectural General Medical Center, and Gifu University Hospital (Gifu, Japan) between January 2010 and December 2022. The database used for our study included the clinical data of all ERCP procedures performed at our institutions between January 2010 and December 2022. The inclusion criteria were age ≥ 75 years and underwent ERCP for CBDS. The exclusion criteria were as follows: post‐endoscopic sphincterotomy (EST), endoscopic papillary balloon dilation (EPBD), or endoscopic papillary large balloon dilation (EPLBD) papilla; acute pancreatitis caused by CBDS; underwent two‐stage endoscopic management—initial biliary drainage alone followed by papillary procedure and stone extraction at a later session—due to severe cholangitis or taking antiplatelet or anticoagulant drugs; and underwent balloon enteroscopy‐assisted ERCP for CBDS on a surgically altered anatomy other than Billroth I reconstruction. Patients were divided into two groups, adjusted to result in no significant differences in patient background using propensity score matching: A (asymptomatic CBDS) and S (symptomatic CBDS).

The Institutional Review Boards of each institution (Gifu Prefectural General Medical Center (Registration number: 929, date of approval: April 25, 2024)) approved this study, which adhered to the tenets of the Declaration of Helsinki. Participant consent was obtained using an opt‐out methodology.

### 2.2 Endoscopic Procedures

ERCP was performed using a side‐viewing endoscope (JF‐260 V or TJF‐260 V; Olympus, Tokyo, Japan). Contrast‐assisted or wire‐guided cannulation was attempted to access the biliary tract. If selective biliary cannulation was difficult, a double‐guidewire technique or pre‐cutting was performed, as appropriate. Following bile duct intubation, cholangiography was performed to evaluate the size and number of CBDS and the bile duct diameter. The duodenal papilla was dilated using EST (Stone Master V; Olympus, Tokyo, Japan), EPBD (Stone Master V; Olympus), or EPLBD (REN; Kaneka Medical Products, Osaka, Japan) for extraction of CBDS. EST is usually performed in a standard manner using a papillotome over the guidewire, with an incision over the roof of the ampulla. When EST was difficult to perform due to a high risk for bleeding, such as in those taking antiplatelet or anticoagulant drugs, undergoing hemodialysis, or decompensated liver cirrhosis, EPBD was performed using a small balloon (up to 12 mm in diameter). However, EPBD alone is known to be associated with a high risk for PEP due to the lack of separation of the bile duct and pancreatic duct openings [10]; endoscopic sphincterotomy plus balloon dilation (ESBD), a procedure in which EPBD is added to a small incision made by EST, was chosen based on the endoscopist's judgment. Furthermore, when endoscopic stone removal was expected to be difficult, such as with large or multiple CBDS, EPLBD was performed using a large balloon (12–20 mm in diameter) with or without preceding EST. Balloon size was selected based on stone size and the diameter of the distal bile duct. The balloon was inflated gradually until the notch disappeared. CBDS were retrieved using a basket and/or extraction balloon catheter. If the CBDS was deemed excessively large for removal, a mechanical lithotripter (ML) was used to crush the stone. When it was uncertain whether the stones had been completely removed or when continuous drainage was required due to acute cholangitis, a biliary drainage tube, including endoscopic nasobiliary drainage (ENBD) and endoscopic biliary stent (EBS), was inserted. Commonly, 5 Fr or 6 Fr ENBD (Gadelius Medical, Tokyo, Japan, or Piolax Medical, Yokohama, Japan) was used. In patients at risk for ENBD self‐extraction, EBS was chosen based on the endoscopist's judgment. Plastic stents (Through & Pass, Gadelius Medical, Tokyo, Japan; or Flexima, Boston Scientific, MA, USA) were used. The ENBD and EBS were removed after clinical symptoms improved, and cholangiography confirmed the absence of residual CBDS.

### 2.3 Study Outcome and Definitions

The primary outcomes included early AEs associated with endoscopic treatment of symptomatic and asymptomatic CBDS. Secondary outcomes included technical success rate and late AEs in both groups. Asymptomatic CBDS were defined as stones with no symptoms or abnormal blood data related to CBDS at the time of ERCP. In contrast, symptomatic CBDS were defined as stones causing obstructive jaundice, acute cholangitis, or elevated liver enzymes. The severity of acute cholangitis was based on the Tokyo Guidelines 18 [11]. AEs associated with the endoscopic procedure were classified according to the lexicon by the American Society of Gastrointestinal Endoscopy [12], distinguishing early AEs (occurring within 2 weeks after ERCP) from late AEs (occurring ≥ 2 weeks after ERCP). PEP was defined as typical abdominal pain in conjunction with serum amylase or lipase levels elevated to more than three times the upper limit of normal [13]. Technical success was defined as complete stone removal during the initial ERCP. Complete stone removal was defined as the absence of filling defects within the bile duct on cholangiography.

### 2.4 Matching Method and Statistical Analysis

Propensity score matching was used to reduce selection bias and potential confounding factors related to baseline characteristics between the two groups. Rigorous adjustments were performed for the following nine factors using propensity score matching analysis: age, sex, use of antithrombotic agents, Billroth I, bile duct diameter, maximum CBDS size, number of CBDS, periampullary diverticulum, and gallbladder status [14]. Propensity score matching analysis was performed based on the following algorithm: 1:1 optimal match using a caliper width of 0.2 in a standard deviation of the logit of the propensity score, with no replacement [15].

Data are expressed as number of patients (n) or median (range). Fisher's exact test was used to compare categorical variables, whereas the Mann–Whitney U‐test was used to compare continuous variables. The cumulative incidence of late AEs was estimated using the Kaplan–Meier method. Cumulative incidence was further compared between the two groups using the log‐rank test. Differences with *p* < 0.05 were considered to be statistically significant. Statistical analyses were performed using EZR version 4.2.1 (Saitama Medical Center, Jichi Medical University, Saitama, Japan) or JMP version 10 (SAS Institute Inc., Cary, NC, USA).

## 3 Results

### 3.1 Patient Selection and Matching

**FIGURE 1:**
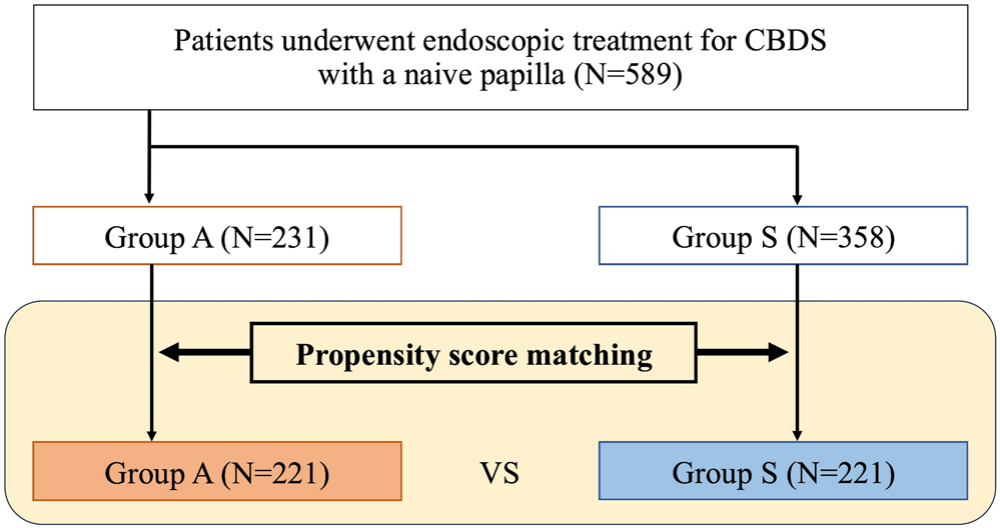
Flow of the study patients. CBDS: Common bile duct stones.

This study included data from 589 patients with CBDS: 231 asymptomatic, 358 symptomatic. After propensity score matching, data were extracted from 442 patients (221 patients in each group) (Figure 1). The baseline characteristics of the overall and propensity score‐matched cohorts are summarized in Table 1. Significant differences were observed between groups A and S in terms of age and use of antithrombotic agents before propensity score matching; however, these differences were well adjusted in the propensity score‐matched cohort. In the propensity score‐matched cohort, group S included 157 patients with cholangitis, 38 with elevated liver enzymes, and 26 with abdominal pain or vomiting.

**TABLE 1:** Baseline characteristics of the overall and propensity score‐matched cohorts.

	Whole cohort			Propensity score matching cohort		
	Group A	Group S	*p*	Group A	Group S	*p*
	(*N* = 231)	(*N* = 358)		(*N* = 221)	(*N* = 221)	
Age, y.o, median (range)	84 (75–99)	85 (75–101)	0.01	84 (75–99)	84 (75–100)	0.93
Sex, Male, *n* (%)	109 (47.2)	162 (45.3)	0.67	104 (47)	104 (47)	> 0.99
Antithrombotic agents, *n* (%)	60 (26)	63 (17.6)	0.01	55 (24.9)	52 (23.5)	0.82
Billroth‐I, *n* (%)	6 (2.6)	8 (2.2)	0.62	5 (2.3)	7 (3.2)	0.77
Bile duct diameter, mm, median (range)	11 (3.6–38)	11 (3–33)	0.61	11 (3.6–38)	11.6 (3–33)	0.11
Maximum CBDS size, mm, median (range)	8 (2–50)	7 (2–47)	0.33	7.6 (2–50)	9 (2–47)	0.13
Number of CBDS, median (range)	2 (1–20)	2 (1–20)	0.37	2 (1–20)	2 (1–20)	0.66
Periampullary diverticulum, *n* (%)	81 (35.1)	147 (41.1)	0.16	80 (36.2)	75 (33.9)	0.69
Status of gallbladder, *n* (%)			0.13			0.43
GB with stone in situ	137 (59.3)	241 (67.3)		134 (60.6)	143 (64.7)	
GB without stones in situ	73 (31.6)	92 (25.7)		69 (31.2)	57 (25.8)	
Status post CCx	21 (9.1)	25 (7)		18 (8.1)	21 (9.5)	
The reason for treatment of CBDS, *n* (%)						
Cholangitis		267 (74.6)			157 (71)	
Mild/moderate/severe		161/92/14			95/54/8	
Elevated liver enzymes		56 (15.6)			38 (17.2)	
Abdominal pain/Vomit		35 (9.8)			26 (11.8)	
The modarity to detect asymptomatic CBDS, *n* (%)						
CT	145 (62.8)			142 (64.3)		
Transbodominal US	47 (20.3)			44 (19.9)		
MRI	24 (10.4)			22 (9.9)		
EUS	15 (6.5)			13 (5.9)		

Abbreviations: CBDS, Common bile duct stones; CCx, Cholecystectomy; CT, Computed tomography; EUS, Endoscopic ultrasonography; GB, Gallbladder; MRI, Magnetic resonance imaging; US, Ultrasonography.

### 3.2 Results and Clinical Outcomes of ERCP

Results of the endoscopic procedures are summarized in Table 2. No significant differences were observed in the use of rectal nonsteroidal anti‐inflammatory drugs (NSAIDs), biliary cannulation method, pancreatic stent, papillary procedure, requirement for ML, or peroral cholangiography between the two groups. However, biliary drainage was performed more frequently in group S than in group A.

**TABLE 2:** Endoscopic procedures of the two groups.

	Group A	Group S	*p*‐value
	(*N* = 221)	(*N* = 221)	
Rectal NSAIDs, *n* (%)	72 (32.6)	60 (27.1)	0.25
Cannulation method, *n* (%)			0.05
Standard	153 (69.2)	176 (79.6)	
Guidewire‐assisted	33 (14.9)	21 (9.5)	
Pancreatic guidewire‐assisted	24 (10.9)	16 (7.2)	
Pre‐cut	11 (5)	8 (3.6)	
Pancreatography, *n* (%)	65 (29.4)	67 (30.3)	0.91
Biliary drainage, *n* (%)			< 0.01
ENBD/EBS/none	48 (21.7)/2 (0.9)/171 (77.4)	91 (41.2)/8 (3.6)/122 (55.2)	
Pancreatic stent, *n* (%)	1 (0.4)	0	> 0.99
Papillary procedure, *n* (%)			0.49
EST/ESBD/EPBD/EPLBD	156 (70.1)/11 (5)/7 (3.2)/47 (21.3)	157 (71)/5 (2.3)/7 (3.2)/52 (23.5)	
ML, *n* (%)	44 (19.9)	57 (25.8)	0.17
EHL with POCS, *n* (%)	3 (1.4)	2 (0.9)	> 0.99

Abbreviations: EBD, endoscopic biliary drainage; EHL, electrohydraulic lithotripsy; ENBD, endoscopic nasobiliary drainage; EPBD, Endoscopic papillary balloon dilatation; EPLBD, Endoscopic papillary large balloon dilatation; EPST, Endoscopic pancreatic sphincterotomy; ESBD, Endoscopic sphincterotomy with balloon dilatation; EST, Endoscopic sphincterotomy; ML, mechanical lithotripter; NSAIDs, Non‐steroidal anti‐inflammatory drugs; POCS, peroral cholangiography.

Clinical outcomes of the two groups are summarized in Table 3. Technical success was achieved in 194 of 221 patients (87.8%) in group A and 187 of 221 (84.6%) in group S (*p* = 0.40). The median number of ERCP procedures performed for complete CBDS removal was 1 (range, 1–4) in group A and 1 (range, 1–3) in group S. The median length of hospital stay was 6 days (range, 3–35 days) in group A and 8 days (range, 3–33 days) in group S. There was no significant difference in the number of ERCP procedures between the two groups (*p* = 0.31), whereas the length of hospital stay was significantly longer in group S than in group A (*p* < 0.01).

**TABLE 3:** Clinical outcomes of ERCP of the two groups.

	Group A	Group S	*p*
	(*N* = 221)	(*N* = 221)	
Technical success, *n* (%)	194 (87.8)	187 (84.6)	0.4
Number of ERCP procedure for complete CBDS removal, times, median (range)	1 (1–4)	1 (1–3)	0.31
Length of hospital stay, days, median (range)	6 (3–35)	8 (3–33)	< 0.01

Abbreviations: CBDS, Common bile duct stones; ERCP, Endoscopic retrograde cholangiopancreatography.

### 3.3 Adverse Events

Of 442 patients, 55 (12.4%) experienced early ERCP‐related AEs, including PEP, bleeding, cholangitis/cholecystitis, and perforation (Table 4). In group A, 29 of 221 (13.1%) patients experienced AEs, whereas in group S, 26 of 221 (11.8%) patients experienced AEs (*p* = 0.77). Among the ERCP‐related AEs, PEP was the most common. The incidence of PEP in group A was 14 of 221 (6.3% [mild, *n* = 11; moderate, *n* = 3]). In contrast, the incidence of PEP in group S was 10 of 221 (4.5% [mild, *n* = 9; moderate, *n* = 1]). There was no significant difference in the frequency or severity of PEP between the two groups (*p* = 0.53). All patients with PEP were managed conservatively. Post‐ERCP bleeding was observed in 12 patients (mild in all 12) in group A and 12 (mild, *n* = 11; moderate, *n* = 1) in group S; the difference, however, was not statistically significant (*p* > 0.99). Bleeding in all patients was well controlled with local therapy using a balloon catheter, injection of hypertonic saline epinephrine, clipping, and a fully covered self‐expandable stent. Post‐ERCP cholangitis/cholecystitis was noted in two patients (mild, *n* = 1; severe, *n* = 1) in group A and three in group S (all mild); the difference was not statistically significant (*p* > 0.99). One patient with severe cholangitis died due to underlying liver cirrhosis or worsening heart failure, whereas the other patient was treated conservatively. Post‐ERCP perforation occurred in only one patient in group A and was treated conservatively with ENBD.

**TABLE 4:** Early ERCP‐related AEs of the two groups.

	Group A	Group S	*p*
	(N = 221)	(*N* = 221)	
Overall, *n* (%)	29 (13.1)	26 (11.8)	0.77
Post ERCP pancreatitis	14 (6.3)	10 (4.5)	0.53
Mild	11 (5)	9 (4.1)	
Moderate	3 (1.4)	1 (0.4)	
Severe	0	0	
Bleeding	12 (5.4)	12 (5.4)	> 0.99
Cholangitis/Cholecystitis	2 (0.9)	3 (1.4)	> 0.99
Perforation	1 (0.4)	0	> 0.99
Others	1 (0.4)	1 (0.4)	> 0.99

Abbreviations: AEs, adverse events; ERCP, Endoscopic retrograde cholangiopancreatography.

**FIGURE 2:**
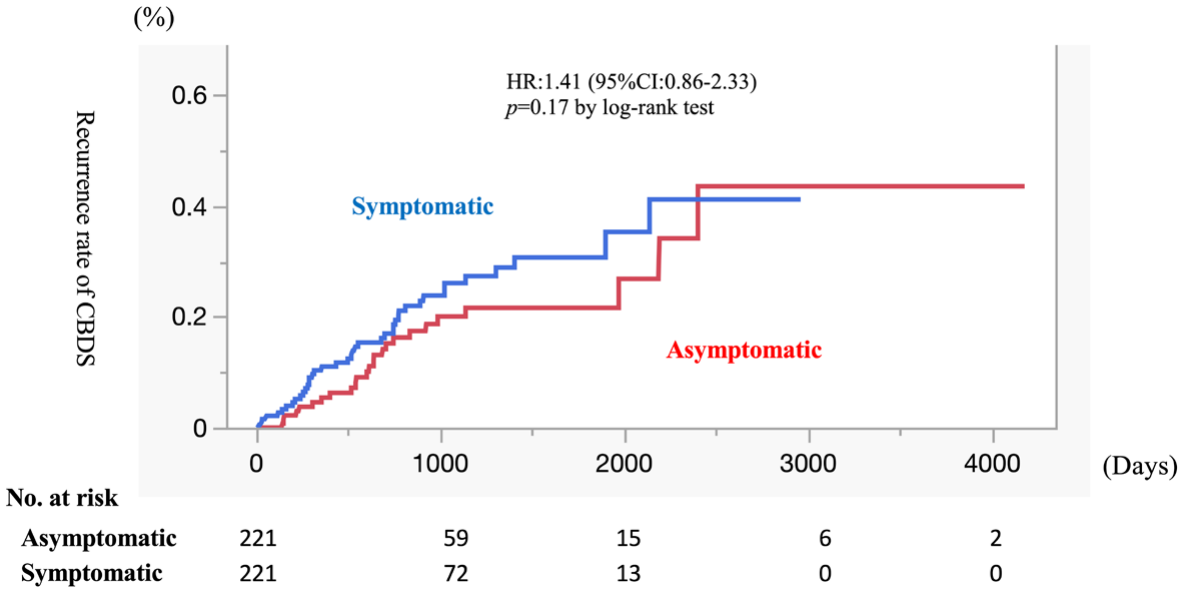
Recurrence rate of CBDS after ERCP. CBDS: Common bile duct stones, ERCP: Endoscopic retrograde cholangiopancreatography, HR: Hazard ratio, 95% CI: 95% Confidence interval.

Late AEs in the two groups are summarized in Table 5. In group A, 32 of 221 (14.5%) patients experienced late AEs, whereas 50 of 221 (22.6%) patients in group S experienced late AEs (*p* = 0.03), with a median follow‐up of 356 days in group A and 614 days in group S. Among these, recurrence of CBDS was the most common. The incidence of recurrent CBDS was 25 of 221 (11.3%) in group A and 41 of 221 (18.6%) in group S (*p* = 0.04). The cumulative incidence of recurrent CBDS according to Kaplan–Meier analysis tended to be higher in group S (hazard ratio 1.41 [95% confidence interval 0.86–2.33]; log‐rank *p* = 0.17) (Figure 2).

**TABLE 5:** Late AEs of the two groups.

	Group A (*N* = 221)	Group S (*N* = 221)	*p*
1‐year AE, *n* (%)	16 (7.2)	24 (10.9)	0.24
3‐year AE, *n* (%)	27 (12.2)	45 (20.4)	0.02
Overall	32 (14.5)	50 (22.6)	0.03
‐CBDS	25 (11.3)	41 (18.6)	0.04
‐Cholecystitis	6 (2.7)	8 (3.6)	0.78
‐Cholangitis	3 (1.4)	3 (1.4)	> 0.99
Follow‐up periods, day, median (IQR)	356 (37–1103)	614 (86–1199)	0.02

Abbreviations: AEs, adverse events; CBDS, Common bile duct stones; IQR, Interquartile range.

## 4 Discussion

### 4.1 Summary

In our study, patients ≥ 75 years old with CBDS were divided into two groups and clinical outcomes were compared: A (asymptomatic CBDS) and S (symptomatic CBDS). Regarding ERCP‐related early AEs, the incidence of PEP was 6.3% in group A and 4.5% in group S, with no significant difference (*p* = 0.53). There were no significant differences in other early AEs (bleeding, cholangitis, cholecystitis, and gastrointestinal perforation) between the two groups. The technical success rate was 87.8% in group A and 84.6% in group S, with no significant difference. The most common late AE was CBDS recurrence (11.3% in group A versus 18.6% in group S [*p* = 0.04]). Kaplan–Meier analysis revealed a tendency toward a higher cumulative incidence in group S.

### 4.2 Risks of ERCP for Asymptomatic CBDS

Asymptomatic CBDS can be fatal if it becomes severe due to complications such as acute cholangitis or pancreatitis; accordingly, the 2021 cholelithiasis guidelines suggest that stone extraction be performed for asymptomatic CBDS [1]. However, ERCP for asymptomatic CBDS is reportedly associated with a higher risk for PEP, and the advantages and disadvantages of its indications remain controversial. Kim et al. compared the clinical outcomes and AEs of ERCP in 32 patients with asymptomatic CBDS and 536 with symptomatic CBDS and reported that the incidence of PEP was significantly higher in the asymptomatic group (12.5% vs. 3.9%; *p* = 0.045) [16]. Similarly, Saito et al. performed a propensity score matching analysis to examine the risk for PEP during therapeutic ERCP for asymptomatic CBDS. They compared outcomes between 158 patients with asymptomatic CBDS and 158 with symptomatic CBDS and found PEP rates of 15.2% and 3.2%, respectively (*p* < 0.001, odds ratio = 5.5) [5]. A possible explanation for the higher incidence of PEP in asymptomatic patients is the higher number with non‐dilated CBD and difficult cannulation; both risk factors for PEP were higher in those who were asymptomatic than in those who were symptomatic [17]. In such patients, pancreatic guidewire cannulation or precut techniques are often required, which are risk factors for PEP [18]. Conversely, the use of rectal NSAIDs and prophylactic pancreatic stenting is effective in preventing PEP [19, 20]. Considering these findings, whether ERCP should be performed in asymptomatic patients with CBDS remains controversial. In our study, factors potentially contributing to difficult deep cannulation, such as CBD diameter and periampullary diverticulum, were adjusted for using propensity score matching. Furthermore, regarding ERCP‐related factors, there were no significant differences between the two groups in biliary cannulation method, distal bile duct diameter, use of rectal NSAIDs, and prophylactic pancreatic stenting, all factors known to influence the risk for PEP; therefore, the incidence of PEP may not differ significantly between the groups. Moreover, the incidence of PEP in the asymptomatic group (6.3%) was lower than that previously reported, possibly due to our study population of elderly patients. In a prospective analysis involving 1284 consecutive ERCPs in patients 20–101 years of age, Casalis et al. reported that advanced age (≥ 80 years) was associated with a significantly lower risk for PEP than in patients 50–79 years of age (1.6% vs. 5.7%; *p* = 0.02) [21]. This may be explained by pancreatic atrophy with aging due to fat infiltration and fibrosis, which leads to reduced exocrine function [22], thus rendering the pancreas less susceptible to mechanical stimulation during ERCP. These findings suggest that ERCP can be safely performed for asymptomatic CBDS in elderly patients due to the lower risk for PEP if appropriate measures are implemented.

In contrast, when adopting a “wait‐and‐see” approach for asymptomatic CBDS, the cumulative incidence of biliary events was 6.1% at 1 year, 11% at 3 years, and 17% at 5 years, suggesting that a certain risk for biliary events exists with this approach [23]. Furthermore, in elderly patients (≥ 75 years of age) who develop CBDS‐related cholangitis, the incidence of severe acute cholangitis (*p* < 0.001), shock (*p* = 0.029), respiratory dysfunction (*p* = 0.002), and renal dysfunction (*p* = 0.006) has been reported to be higher than that in non‐elderly patients [24]. This process may lead to a decline in the activities of daily living and deterioration of cardiopulmonary function. These findings support the active consideration of ERCP for asymptomatic CBDS in elderly patients, before the onset or aggravation of biliary events.

In addition, the recurrence of CBDS tended to be higher in group S. To date, dilated common bile duct (≥ 15 mm), periampullary diverticulum, cholelithiasis, multiple CBDS, the use of mechanical lithotripters, biliary stenting, and advanced age have been reported to be risk factors for recurrence of CBDS [25]. Another study reported that biliary tract infections may affect the recurrence of CBDS due to impaired biliary tract function [26]. During emergency ERCP for symptomatic CBDS, particularly when performing a one‐stage procedure, it is sometimes difficult to perform adequate cholangiography and evaluate residual stones to prevent the deterioration of the patient's condition, which may contribute to the recurrence of CBDS [27]. Due to these factors, symptomatic CBDS may have a higher recurrence rate after ERCP compared with asymptomatic CBDS, and it has also been reported that once a patient experiences recurrence, the rate of subsequent recurrence increases [28]. Therefore, planned stone extraction for asymptomatic CBDS in elderly patients is clinically significant because it may prevent severe biliary AEs and improve quality of life and prognosis.

### 4.3 Treatment Strategy for Asymptomatic CBDS Based on Our Study and Previous Reports

In our study, ERCP for asymptomatic CBDS in elderly patients showed no significant difference in early AEs, including PEP, and technical success rate compared with symptomatic CBDS, whereas for late AEs, the recurrence rate of CBDS tended to be higher in symptomatic patients. Wait‐and‐see management of asymptomatic CBDS is associated with a certain frequency of biliary events, especially in elderly patients who are at risk for severe complications and potentially fatal outcomes due to multiple comorbidities and impaired cardiopulmonary function. These findings suggest that it is reasonable to consider active ERCP intervention in elderly patients, even in those with asymptomatic CBDS, after implementing adequate measures against AEs due to the high risk for symptomatic or recurrent events.

### 4.4 Limitation

Our study had several limitations. First, there may have been residual confounding variables, even though the baseline characteristics were well adjusted using propensity score‐based cohort matching. Second, the incidence of AE rate in the asymptomatic group may have been underestimated because our study did not include the patients with CBDS who were followed up without ERCP procedure due to high risk of AE. Third, although our findings support guideline‐based ERCP for asymptomatic CBDS, a direct comparison between the ERCP intervention and wait‐and‐see approach was not performed; therefore, it cannot be uniformly recommended, and particularly caution is required for elderly patients with poor performance status. Fourth, complete stone removal was defined as the absence of filling defects within the bile duct on cholangiography in our study; however, there was no standardized protocol for biliary drainage, and residual stones might not have been accurately assessed. As a result, it cannot be ruled out that some patients of recurrent CBDS may have included residual stones. Fifth, because this was a retrospective study, there was no standardized follow‐up protocol, and the follow‐up duration differed significantly between the two groups, which may have contributed to the differences in late AE rates. Finally, the follow‐up period may have been insufficient to evaluate late AEs that occurred > 5 years.

## 5 Conclusion

In conclusion, endoscopic transpapillary stone extraction in asymptomatic elderly patients may be beneficial because these individuals are at a higher risk for severe biliary complications due to underlying diseases.

## Author Contributions

Kota Shimojo and Akinori Maruta conceived, designed, and analyzed the study. Yuhei Iwasa, Mitsuru Okuno, Kensaku Yoshida, Shinya Uemura, Keisuke Iwata, Tsuyoshi Mukai, Takuji Iwashita, and Masahito Shimizu approved medical approaches in the publications. Kota Shimojo and Akinori Maruta contributed to the writing of the manuscript. Akinori Maruta took the correspondence. All authors read and approved the final manuscript.

## Funding

The authors have nothing to report.

## Ethics Statement

Approval of the research protocol by an Institutional Review Board: Gifu Prefectural General Medical Center.

## Consent

Participant consent was obtained through an opt‐out methodology.

## Conflicts of Interest

The authors declare no conflicts of interest.

## Data Availability

The data that support the findings of this study are available on request from the corresponding author. The data are not publicly available due to privacy or ethical restrictions.
